# A Research Strategy Case Study of Alcohol and Drug Prevention by Non-Governmental Organizations in Sweden 2003-2009

**DOI:** 10.1186/1747-597X-6-8

**Published:** 2011-04-14

**Authors:** Charli Eriksson, Susanna Geidne, Madelene Larsson, Camilla Pettersson

**Affiliations:** 1 School of Health and Medical Sciences, Örebro University, S-701 82 Örebro, Sweden

## Abstract

**Background:**

Alcohol and drug prevention is high on the public health agenda in many countries. An increasing trend is the call for evidence-based practice. In Sweden in 2002 an innovative project portfolio including an integrated research and competence-building strategy for non-governmental organisations (NGOs) was designed by the National Board of Health and Welfare (NBHW). This research strategy case study is based on this initiative.

**Methods:**

The embedded case study includes 135 projects in 69 organisations and 14 in-depth process or effect studies. The data in the case study has been compiled using multiple methods - administrative data; interviews and questionnaires to project leaders; focus group discussions and seminars; direct and participatory observations, interviews, and documentation of implementation; consultations with the NBHW and the NGOs; and a literature review. Annual reports have been submitted each year and three bi-national conferences Reflections on preventions have been held.

**Results:**

A broad range of organisations have been included in the NBHW project portfolio. A minority of the project were run by Alcohol or drug organisations, while a majority has children or adolescents as target groups. In order to develop a trustful partnership between practitioners, national agencies and researchers a series of measures were developed and implemented: meeting with project leaders, project dialogues and consultations, competence strengthening, support to documentation, in-depth studies and national conferences. A common element was that the projects were program-driven and not research-driven interventions. The role of researchers-as-technical advisors was suitable for the fostering of a trustful partnership for research and development. The independence of the NGOs was regarded as important for the momentum in the project implementation. The research strategy also includes elements of participatory research.

**Conclusions:**

This research strategy case study shows that it is possible to integrate research into alcohol and drug prevention programs run by NGOs, and thereby contribute to a more evidence-based practice. A core element is developing a trustful partnership between the researchers and the organisations. Moreover, the funding agency must acknowledge the importance of knowledge development and allocating resources to research groups that is capable of cooperating with practitioners and NGOs.

## Introduction

Alcohol and drug prevention is high on the public health agenda in most countries. The national initiatives differ, although action plans have been proposed by international organizations such as WHO [1]. Moreover, there is an increasing demand for evidence-based alcohol and drug prevention, causing an increased emphasis on research for prevention, an emphasis that this field shares with health promotion, prevention in general, and social work [2-8]. This means that prevention research needs to move "from basic to more and more applied research; from descriptive hypothesis-generating pilot studies to full-fledged, methodologically sophisticated, hypothesis-testing studies; from smaller to larger samples for testing; from greater to lesser control of experimental conditions; from more artificial 'laboratory' environments to real-world geographically defined communities; from testing the effects of single intervention strategies into more complex studies of multiple strategies integrated into intervention systems; and from research-driven outcome studies to 'demonstration' projects that evaluate the capacity of various types of communities to implement prevention programs based on prior evaluations" [[9], p 183]. It has also been more than 10 years since Nutbeam [10,11] noted the gap between the need for knowledge and the priorities among researchers.

Many years have passed since these recommendations, but still the gap between evidence and practice has not been bridged despite important achievements in implementation research [12], designs for effectiveness and translation research [13], and a series of initiatives regarding the evidence-practice gaps [14-22]. The call for more practice-based evidence is a challenge for policy-makers, practitioners, researchers, and funding agencies [17,23]. In several countries research on alcohol and drug issues has been incorporated into addiction research centres [24-28]. For many years much addiction research has been the product of specialized research centres rather than the contribution of standalone scientists. Moreover it is the specialist centres, in collaboration with the national funding agencies, which today assert leadership, set agendas, and help determine standards [24]. However, a common element in the missions of these centres is monitoring the substance use in the population, its causes, and courses, while prevention research is not high on the agendas. Furthermore, the establishment of national centres demonstrates the political administration's emphasis on scientific, evidence-based policies, but at the same time demonstrates the view that credible research is best performed within independent scientific bodies [26].

In Sweden in 2002 an innovative project portfolio for non-governmental organisations (NGOs) was designed by the National Board of Health and Welfare (NBHW). This included an integrated research and competence-building strategy to strengthen alcohol and drug prevention. This case study aims to describe and analyse this initiative.

### AD prevention in Sweden - legislation, national action plans, resources, and actors

Sweden has a long tradition of a restrictive alcohol policy [29]. The temperance movement became a powerful actor in the Swedish alcoholic beverage policy [30]. Moreover, Sweden is one of the few countries in Europe with a narcotics policy that aims to create a society entirely free of illicit drugs [31].

The overall goal of the Swedish action plan on alcohol and narcotics is to promote public health by reducing the medical and social harm caused by alcohol and to create a drug-free society. The strategy for achieving this goal with regard to alcohol is to reduce the total consumption and prevent harmful drinking, taking into account differences in living conditions among boys, girls, men, and women. Six priority sub-goals have been adopted: alcohol should not be consumed in transport contexts, at workplaces, or during pregnancy; children should grow up in an alcohol-free environment; the age of alcohol debut should be postponed; drinking to point of intoxication should be reduced; there should be more alcohol-free environments; and illicit alcohol should be eliminated. The sub-goals in the action plan on narcotics are to reduce recruitment to drug abuse, induce people with substance abuse problems to give up their abuse, and to reduce the supply of drugs. Interventions targeting children, adolescents, and parents are of high priority [32].

Swedish alcohol policy is based on a combination of taxed-based price controls and the alcohol retail monopoly in order to limit the availability and accessibility of alcohol [32]. There is strong evidence for the preventive effects of an alcohol retail monopoly [33,34] and high prices on alcohol are regarded as one of the most effective ways of reducing total alcohol consumption and alcohol-related problems [35]. When Sweden entered the EU in 1995, the conditions changed and Sweden could no longer have an independent alcohol policy. For example, the availability of alcohol increased as a result of changed rules for private import, and alcohol taxes had to be adjusted. The numbers of alcohol shops as well as their opening hours have also increased remarkably since 1995 [29]. The increased movements across borders have also had an influence on the illicit drugs market. Almost all narcotics that are consumed in Sweden have been produced outside the country. A well-developed international collaboration is therefore of high importance for the limitation of illicit drugs in Sweden [32].

An effective alcohol and drug policy also requires national coordination. The Swedish government has established a national council for alcohol, narcotics, doping, and tobacco. The council consists of members of public authorities, civil society, and researchers, and is led by the State Secretary of the Ministry of Health and Social Affairs. The council is commissioned to advise the government on issues about alcohol, drugs, doping, and tobacco and to present information about research results [36].

There is a need for the different sectors in society to increase and deepen their cooperation for an effective prevention of the use of alcohol, tobacco, and drugs. In the Swedish action plan on alcohol and illicit drugs as well as in the government bill for public health the importance of the voluntary sector is emphasized [36,37]. In the latter document, *A renewed public health policy*, it is stated that cooperation between the state and the voluntary sector should be expanded and that the conditions for the voluntary sector's work should improve [37]. An agreement about the relations between the government, the voluntary sector in the social setting, and the Swedish Association of Local Authorities and Regions has recently been developed through a dialogue between the parties. The dialogue is another way for the government to call attention to the voluntary sector and to its ambition to strengthen the sector and improve its conditions. The goal of the agreement was to strengthen the independence of the voluntary sector as moulders of public opinion and to support the development of public medical service carried out by the voluntary sector [38]. The Swedish voluntary sector has a long tradition of alcohol prevention, especially the temperance movements [39].

### NGOs in Sweden

The Swedish voluntary sector is both different and similar to those in other countries. A major difference lies in its history in that, for instance, as early as in the 16th century the responsibility for health and care was organized under the state instead of in the regime of the church. In parts of Europe the church still is an active actor in health and care [40,41]. Also, popular mass movements have played an important role in the development of Swedish society [41,42]. The Swedish voluntary sector is as large as in other industrialized countries, although quite different in character. It is dominated by organizations in the cultural and recreational field, mainly sports organizations. Since the early 1990s the Swedish voluntary sector has expanded, particularly in the two areas of culture and recreation, as well as in the area of social care [43]. It can also be called membership-based; almost everyone in Sweden is a member of some organization. Because of these differences in history and structure in different societies, the voluntary sector plays different roles. In Sweden, NGOs are more of a complement then a substitute for state programs, and have an important role as forerunners and innovators [44].

Previous research has shown that the Swedish voluntary sector was highly dependent on public financing, which is partly correct. Looking at the entire sector together, about 30 percent of its financing comes from government funding. However, within the health care and social service sector, public financing stands for more than 70 percent. That is quite high in comparison with other European countries, but not the highest [45].

### Support to NGOs today

#### Organizational grants

The National Board of Health and Welfare (NBHW) has a government commission to administer the grants to national organizations for the disabled, the elderly, and relatives of elderly persons; to national organizations in the social setting; and to national and local organizations. For the moment this amounts to about 300 million SEK to about 100 organizations. Also the Swedish National Institute of Public Health has funds to distribute to NGOs or to other organizations working together with NGOs. The Swedish State Inheritance Fund is also a possible source of funding for NGOs. They administer over 300 million SEK a year to provide grants to NGOs working with children, youth, and the disabled. In addition other governmental agencies such as the Swedish National Board for Youth Affairs also support NGOs.

#### Project grants

In the late 1990s a new system of awarding grants to NGOs in the arenas of alcohol and narcotics, vulnerable children and their families, and violence against women was prepared. The previous systems were from the late 1970s and early 1980s, and during the 1990s many investigations recommended a better, more structured follow-up and evaluation of the NGOs' work. One new idea that emerged during the 1990s was increased performance management, that is, the need to point out achieved results and effects of different activities. It was emphasized that the government should not interfere with the running of the organizations but does have the duty to monitor the use of the grants. There was also a desire that renewal efforts and collaborations should be encouraged and supported.

In the late 20th century grants were awarded through the Swedish National Institute of Public Health (with money from the Swedish State Inheritance Fund) to a number of alcohol and drug prevention projects. A final report and an internal evaluation were required from the applicants. There was also an external evaluator, who conducted an evaluation of 11 projects focusing on their working processes [46]. Among the lessons learned from this evaluation were that the way of working should be characterized by frequent contacts and dialogue between the funding agency and the project, and also by supervision. The evaluation report also suggested that the support to the project leaders should be reviewed with regard to the possibility of different types of need-based support. Moreover, the short-term thinking in the funding of these kinds of projects was not in line with the needed time-frame.

### Setting the Scene: NGO strategy for alcohol and drug prevention

**Figure 1:**
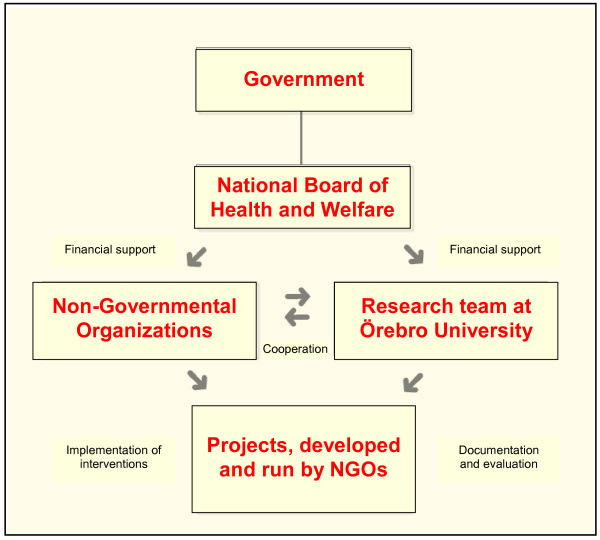
**Integrated research and development for NGO alcohol and drug prevention**.

Non-governmental organizations have received grants from the NBHW to conduct alcohol and drug preventive work in a special venture since 2003 [47]. This initiative is part of the national plan of action to prevent alcohol-related harm and the national plan of action against narcotics and comes from the Ministry of Health and Social Affairs. The working committee, which decides who will get funding, consists of members of the NBHW, the Swedish National Institute of Public Health, and the Swedish National Board for Youth Affairs (previously members of the Swedish Alcohol Committee and the Swedish National Drug Policy Coordinator were included). The working committee, after consulting the research team at Örebro University, also decides which projects will be studied in-depth. NBHW's initiative represented a new way of thinking. One point of departure was to create a project portfolio with a broad combination of organizations to mobilize many forces in the alcohol and drug preventive work. The initiative also contains supervision for the project leaders, competence support through regular meetings for project leaders, and an integrated Research & Development (R&D) investment (Figure 1).

### Need for knowledge building and learning

There is an increasing trend towards promoting evidence-based public health initiatives. International expert committees have presented the state of science with regard to alcohol prevention: Alcohol Control Policies in Public Health Perspectives [48]; Alcohol Policy and the Public Good [49]; and Alcohol: No Ordinary Commodity -Research and Public Policy [33,50]. National authorities have presented reviews presenting evidence for practitioners and politicians [51-53]. However, there are important knowledge gaps to be filled. Among these is the lack of effectiveness studies where the external validity is high. If we want to see more evidence-based practice we need more practice-based evidence [54]. This means an improved emphasis on cooperation between researchers and practitioners [10].

A comprehensive perspective on the concept of knowledge, including scientific and practical knowledge as well as practical wisdom, is needed. Scientific knowledge about alcohol and drug issues needs to be complemented with knowledge about methods for alcohol and drug prevention. As in other public health fields, ethical issues and practical wisdom are important [55]. Moreover, the science, craft, and art of implementation are of utmost importance. There are many reasons besides practicalities that are significant for the implementation of programs [7,56]. In a recent review, 23 different factors were found that were of importance for the degree of the implementation [12] and that also have a great impact on the program effects.

Research on alcohol and drugs has often been organized in special research institutes, which often focus on basic research on alcohol and drugs [24-28]. This basic research is related both to basic biomedicine as well as social and behavioural studies. Another activity, which has been accorded great prominence, is the monitoring of alcohol and drug use in the population in general as well in different groups. Intervention research has been given less prominence in these often national research institutes. However, the national agencies, such as the Swedish Institute of Public Health, have been involved in the evaluation of different intervention projects. So far research on NGO-driven alcohol and drug prevention has been almost completely lacking. Research has been a more or less exclusive activity for the university. However, this has been based on the trust in the impartiality and objectivity of the university-based researchers. The downside of this position is that this type of research may lack the necessary cultural awareness and insights necessary for a proper understanding of basic factors for successfully planning intervention programs as well as evaluating research efforts. In other words the roles of the researcher in intervention studies need to be addressed. In a recent study, Holmila et al. [57] outlined three different positions for researchers in community intervention studies. The researcher can be an external observer, not taking part in the preventive activities - acting as an *unobtrusive observer*. The researcher assumes no responsibility for the design or implementation of the projects but acts as an independent conductor of process evaluation and observer of project outcomes. Another position is to be a *researcher-as-technical advisor*. In this role the researcher has responsibility for evaluation but also takes the responsibility for providing scientific advice on effective preventive strategies if asked for [58-60]. This could include training and technical assistance to the projects. Progress reports on findings as well as results from different on-going studies can be presented to the practitioners, which may use this information as they desire. A third type is *researcher-as-designer*, where the project is designed by the research team in partnership with the practitioner. The researcher is an active participant in project planning as well as the process of carrying it out and evaluating the effects. This approach is particularly useful when the goal is to test one or more designed prevention strategies under as close to optimal conditions as possible. Examples of such in Sweden are the STAD Project in Stockholm [61] and the Trelleborg Project [62]. The Örebro Prevention Program is an example of a program where all parts of the process were in the hands of the researchers [63].

## Aims

The present paper aims to describe and analyse alcohol and drug prevention supported by the NBHW and implemented by NGOs in Sweden during 2003-2009 with a special emphasis on research and development for an evidence-based practice. The case study analyses also the integrated research strategy and its main components.

Three research questions will be addressed:

1. Which types of organizations and projects have received grants from the NBHW for AD prevention?

2. What types of research and development activities for an evidence-based practice have been included?

3. How can a trustful partnership develop between practitioners, national agencies, and researchers?

## Methods and materials

### Case Study Approach

A case study method was chosen as the intention was to understand a real-life phenomenon in depth and the contextual factors were highly pertinent to the study [64]. This method investigates according to Yin contemporary phenomenon in depth and within its real-life context, especially when the boundary between the phenomenon and context are not clearly evident. Moreover, the case study approach copes with the situation such as in this case in which there will be more variables of interest than data, which leads to the need for multiple sources of evidence, with data needing to converge in a triangulating fashion. Furthermore, benefits from the prior development of theoretical propositions to guide data collection and analysis [64]. The present research strategy case study is on an organisational level. It studies a social process in a situation in which we have little knowledge of the phenomenon, integration of research in alcohol and drug prevention run by NGOs. Case studies as a main research strategy are selected as this is a unique case in Sweden, the impossibility to isolate the process and the intention is to combine research and action [65].

An embedded single-case design was chosen for the study. All the projects run by the NGOs are seen as embedded units of analysis in the study with special emphasis on the fourteen in-depth studies.

### Participants

The embedded case study includes 135 projects in 69 organisations and 14 in-depth process or effect studies. The participants in this research strategy case study are the NGOs applying for funding to the NBHW and especially those NGOs that have received funding during 2003-2009. The project leaders and managers in the NGOs as well as the members of the different target groups are also participants in this study. Moreover, staff at the NBHW as well as other stakeholders is included.

### Case study questions

When the research program started a set of overall research questions were developed. In this paper the focus is in one of these, how can a trustful partnership for practice-based research be developed? Additional questions concerns: the role as a project leader in NGOs, the impact of competence development, methods for documentation of project development, and the added value of running projects in NGOs.

### Case study protocol

A plan for the research and development activities was developed the first year and amended each year after the completion of the annual report to the NBHW. This plan consisted of several parts relating to the overall activities as well as the different in-depth studies. Notes were taken at meetings and as part of the strategy a series of presentations as progress reports were given to, project leaders, NGOs and the NBHW.

### Development of a Case Study Database

In the present study a broad range of methods was used in the data collection. This includes six types of data.

#### Administrative data

The applications from the NGOs to the NBHW as well as the funding decisions were the initial data, which was complemented by bi-annual as well as annual progress reports from all funded projects. These reports, which were submitted following a format developed by the research team, gave information on implementation and goal achievement as well as reflections on barriers and facilitating factors. The research team introduced this approach at a meeting with the project leaders, and this reporting resulted in an annual report to the NBHW on the progress of the alcohol and drug prevention projects run by the NGOs.

#### Interviews and questionnaires to project leaders

Data was collected from project leaders and their organizations in the years when funding was received from the NBHW. In 2003, 2005, 2007, and 2009 all project leaders were invited to respond to a questionnaire containing questions on being a project leader in a non-governmental organization. If the same project leaders were responsible for a project for more than one year they responded to more than one questionnaire. Most of those who answered the 2003 questionnaire also answered the 2005 questionnaire, due to the fact that many of those projects receiving funding in 2003 also were being funded in 2005. In total, 84 persons participated in the questionnaire study over the years. Of these, 38 project leaders answered the questions more than one year.

#### Focus group discussions and seminars

Thematic discussions were held as a part of the meetings with the project leaders. These highlighted special issues related to the practice of alcohol and drug prevention. Moreover, a series of joint seminars with NGOs and the research team have been held at national and organizational conferences focusing on different projects.

#### Direct observations, participatory observations, interviews, and documentation of implementation of the in-depth studies

The research team collected information by a variety of methods during the planning, implementation, and evaluation of the in-depth studies. Part of this data has been used in the analysis, resulting in separate reports and scientific publications. However, in this context more process-related data will be used to give insights into the development of the partnerships between researchers and practitioners.

#### Consultation with the NBHW and the NGOs

In the present paper information retrieved during the management of the NBHW support to the NGOs will be an additional source of information. Regular meetings have taken place with the steering committee and the senior administrative officer, who have been the same persons during all years. The consultations with the NGOs were more intense for those organizations selected for in-depth studies, but several meetings have also taken place with other organizations. Apart of the in-depth studies was feedback on preliminary results from different studies; this never radically changed the interpretation of results but did add valuable information.

#### Literature review

A systematic review of the research strategies for alcohol and drug prevention has been carried out as an integral part of the research program. A number of publications related to collaboration between researchers and practitioners were found. Special thematic sections and series have been looked for. Among the key words are addiction research centre, alcohol and drug research, preventive research, practice-based research, and evidence-practice gaps.

### Analytical methods

The analytical approach in this case study follows a common strategy used in research programs: to start with the ordinary preventive activities and then study what is happening [66,67]. Using a naturalistic approach, which is always practice-based, it has been important to let different actors and stakeholders into the knowledge-building program. This also has implications for the selection of research and evaluation methods, given a need for mixed-method approaches [68-70]. In studies of effects, quantitative approaches are essential, but important contributions can be achieved if qualitative studies are also included [71,72]. The mixed-methods approaches have been developed for some of the more extensively studied programs, which also will be included in doctoral dissertations [73,74].

The analysis starts with a quantitative description of the investment in NGOs by agencies awarding grants and an analysis of which organizations and projects that were supported. The types of organizations are analysed with regard to their main focus or mission. Then the investment in research is described including an overview of the participants in different empirical studies using a range of data collection methods. This includes a description of how the embedded units, the project in the NBHW portfolio, have been documented and presented in annual reports using a format for the written reports based on questions and answers in the case study database [64]. The two types of in-depth studies are briefly presented: effect studies and studies of process and implementation.

An analysis of the experiences of cross-project comparisons as well as using the multiple sources of evidence in the case study database follows. The different measures in the research program was developed in order to foster a trustful partnership is then presented. These measures were assessed by all project leaders in the annuals reporting to the research team, which reviewed the content of the research strategy each year in the annual report to the NBHW. The implementation of the research strategy with regard to evaluation initiatives together with the NGOs as well as in-depth studies was carefully documented over the years and used as indicator for developing a research partnership with the NGOs. In this case study the focus is on the implementation of the research and evaluation efforts and not on the outcome of the alcohol and drug prevention program. This has been reported in other publications [47]. The different types of data and perspectives included in the case study database are used for triangulation and finding key elements and mechanisms in the research strategy. In this case study a mixed-methods approach means parallel mixed data analysis, i.e. parallel analysis of qualitative and quantitative data from different sources. Moreover, integrated mixed data analysis also occurs in the analysis of the project portfolio and subsequent development of research initiatives. To grasp the complexity and inclusiveness of integrated methods the term inference has been proposed as the last and most important stage of research [70]. The inference process consists of a dynamic journey from ideas to results in an effort to make sense of data. In our case study the regular project leader meetings as well as the preparation of the annual evaluation and reporting to the NBHW are key activities in this process of drawing inferences. Key concepts in an integrative framework are inference quality, which is related to design quality, interpretative rigour, and inference transferability.

## Results

The results will be presented according to the three research questions. The calls for applications resulted in many proposals from many different organizations for a variety of projects engaging many project leaders.

### Investing in the NGOs - Allocation of Grants 2003-2009

Since 2003 10-15 million SEK per year have been administered to this special venture (Table 1). The government's decisions have over the years differed somewhat according to which target groups are being specially addressed in the calls for grant applications. For the first period, which was a two-year period, the call was broad. For the second period, 2005, the main part of the grants went to projects from the earlier period. From 2006 to 2009 the target groups have been children, youth, young adults, and the workplace according to the national action plans. It has also been emphasized that the projects would be new or in the process of expanding existing activities.

**Table 1:** Grants, number of applications, and number of project grants 2003-2009.

Year	Total grants, in millons of SEK	Number of applications	Number of project grants
2003-2004	25	176	31

2005	10	134	24

2006	15	182	37

2007	15	117	39

2008	15	156	48

2009	15	101	40

Total	95	866	219

About one in four applications were awarded a grant. The amount of funds provided to NGOs varies. The minimum amount of funding for one year was 40,000 SEK and the largest amount was 1,200,000 SEK. Many organizations have been funded for several years. Over the years 2003-2009 the NBHW has in total apportioned about 80,000,000 SEK to the NGOs. In addition a yearly grant has been awarded to an integrated Research & Development (R&D) program as well as funds for administration and information activities. The total allocation from the NBHW has been 95,000,000 SEK, covering 135 projects in 69 organizations funded during these years.

The projects differed in size. Table 2 presents these 219 project grants over the years 2003-2009. The reason for this lower number of project (135) is that 50 projects have been funded over more than one year, 26 projects over two years, 17 projects over three years, and two projects over four and five years each. The first period, which covered two years, had the highest number of large projects. Moreover, the number of funded project has increased between 2005 and 2008 and the number of large projects has remained relatively stable since 2005.

**Table 2:** Overview of NBHW funding/project grants 2003-2009.

Grants, in thousands of SEK	2003 2004	2005	Year 2006	2007	2008	2009	Total
**Total**	**21,060**	**9,110**	**11,585**	**12,134**	**13,862**	**12,670**	**80,121**

< 100	0	1	4	2	6	1	14

100-199	4	6	8	12	9	10	49

200-299	6	7	7	4	10	5	39

300-399	1	2	5	7	8	11	34

400-499	5	1	6	8	7	4	31

≥500	15	7	7	6	8	9	52

Projects	31	24	37	39	48	40	**219**

### Organizations and projects

**Figure 2:**
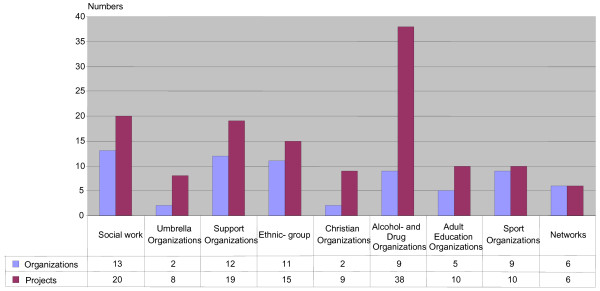
**Organizations and projects in different types of organizations according to main objectives**.

The strategy to involve a broad range of organizations has been successful. In Figure 2 the 69 organizations and the 135 projects are presented according to type of organization. The largest number of projects were run by the nine alcohol and drug organizations. More than half of these projects were run by the Swedish temperance organisation IOGT-NTO (24 of 38 projects) amounting to 15 million SEK. The majority of these were small one-year projects, except for two programs where effect studies were conducted by the research team and the organization jointly. Between 15 and 20 projects were run by organizations focusing on social work, assistance, and ethnic groups. About 10 projects were run by sports, adult education, and religious organizations respectively. Furthermore, 14 projects were set up by two umbrella organizations each consisting of a number of member organizations.

The projects have different primary target groups for their activities. A majority of the projects have children or adolescents as target groups. Some of these projects are focused on young girls with the aim of promoting self confidence and a positive self image. Sports organizations have been developing alcohol and drug policies including anti-doping initiatives. Projects run by ethnic groups have as their target group members of their organizations including children, adolescents, and parents. A few projects have the workplace as the arena for intervention.

During the first years, three community-based projects were funded. These aimed to reduce drugs in two parts of Stockholm and the island Gotland. The strategy included a range of activities and collaboration with different actors. A broad membership in the organizations seems to be important for the sustainability of the community-based prevention.

Only one project has reduction of availability as its focus. This project focused on following up the alcohol legislation concerning the sale of beer to minors in Sweden [75] and the effect of different strategies to influence shops to comply with the law [74].

Internet has a great potential in promotion and preventive work [76]. The majority of the organizations have their own website on the Internet and about one third have a project-specific site. The organizations used information technology as a source of health information in three projects, as an intervention medium in four, for professional development in two, and as a research instrument in one project. The use of e-screening as a tool for drug prevention is studied by researchers at Karolinska Institute. There are still very few scientific evaluations of the use of Internet in drug prevention [77].

Basic characteristics of the project leaders in the alcohol and drug prevention projects are given in Table 3. All four years the proportion of women was larger than men; about two of three project leaders were women. Most of the project leaders belonged to the age group 41-50 years in the early periods (2003/2005) while in the later periods (2007/2009) an increased proportion of the project leaders were 50 years or older. Moreover, nearly one in ten project leaders was 30 years or younger. Many of the project leaders in volunteer work were members of the organization before being appointed project leaders (Table 3). In 2003 eight of ten project leaders were members compared with four of ten in 2007 and 2009. Nearly half of the project leaders were also doing volunteer or non-paid work in the organization. No gender differences were found in the prevalence of non-paid work.

**Table 3:** Descriptions of Project leaders, per cent (n).

Year	2003	2005	2007	2009
Participants	(41)	(35)	(27)	(26)

Women	56 (23)	60 (21)	52 (14)	62 (16)

Men	44 (18)	40 (14)	48 (13)	39 (10)

≤ 30 years	10 (4)	9 (3)	12 (3)	8 (2)

31-40 years	24 (10)	28 (9)	27 (7)	19 (5)

41-50 years	39 (16)	41 (13)	27 (7)	19 (5)

≥ 51 years	27 (11)	22 (7)	35 (9)	54 (14)

Prior member	82 (31)	-	44 (12)	46 (12)

Unpaid work	49 (19)	-	42 (10)	46 (12)

### Investing in Research and Development

A research and evaluation strategy was developed by the research team at the School of Health and Medical Sciences, Örebro University. This strategy rests on collaboration with the NGOs through regular meetings with all project leaders, development of systematic documentation of project objectives, activities, and results, annual reports to the NBHW, and biannual national conferences Reflections on prevention (2006, 2008, and 2010). The role of the researchers can most closely be characterized as *researchers as technical advisors*. In some projects the researcher had the position of an unobtrusive observer -for instance in following up some projects in which no longitudinal data collection was included. In addition, in no project did the researcher have the position of *researcher as designer*. Moreover, separate competence development and discussion of evaluation studies were conducted with a smaller number of organizations. The steering committee at the NBHW also decided, after consulting the research team, on a number of in-depth studies. Fourteen such studies were included in the funding from the NBHW (Table 4). The research team was also involved in three additional studies funded by other sources. These studies focused on policy development in the Swedish Football Confederation, evaluation of regional collaboration against illegal alcohol, and alcohol prevention in Novgorod, Russia.

**Table 4:** Evaluation studies 2003-2009.

	2003-2004	2005	2006	2007	2008	2009
**Hassela solidaritet**: Peer Support in School	NBHW, ÖU	NBHW, ÖU	NBHW, ÖU	ÖU		

**IOGT-NTO Centro**: Parental Support		NBHW, ÖU	NBHW, ÖU			

**IOGT-NTO**: Parents Together			NBHW, ÖU	NBHW, ÖU	NBHW, ÖU, E	ÖU, E

**IOGT-NTO**: Strong and Clear	NBHW, ÖU	NBHW, ÖU	NBHW, ÖU	NBHW, ÖU	ÖU	ÖU

**IOGT-NTO**: Dare/Young and King	NBHW, ÖU	NBHW, ÖU	ÖU			

**IOGT-NTO:s Juniorförbund**: Junis sisters			NBHW, ÖU	NBHW, ÖU	ÖU	ÖU

**KSAN**: About small things	NBHW, ÖU	NBHW, ÖU	ÖU	NBHW, ÖU		

**Makalösa föräldrar**: Single Parent with Teenagers	NBHW, ÖU	NBHW, ÖU	NBHW, ÖU			

**Motgift Gotland**: Antidote Gotland	NBHW, ÖU	NBHW, ÖU	ÖU			

**The National Federation SMART**: SMART Västernorrland	NBHW, ÖU	NBHW, ÖU	ÖU			

**Söder mot narkotika**: Södermalm against Drugs	NBHW, ÖU	NBHW, ÖU	ÖU			

**The Swedish Ice Hockey Association**: School Ambassadors	NBHW, ÖU	NBHW, ÖU	NBHW, ÖU			

**UNF**: Folk Beer Project	NBHW, ÖU	NBHW, ÖU	ÖU	ÖU		

**Verdandi Tensta Rinkeby**: Get Safe in Tensta Rinkeby	NBHW, ÖU	NBHW, ÖU		E, ÖU	ÖU	

NBHW = support from NBHW; ÖU = Research Activity; E =, External support from other funders.

This set of studies included systematic collection of data from children, parents, and actors in projects. A description of the empirical studies carried out between autumn 2003 and spring 2009 is given in Table 5. Different methods, including questionnaires, personal interviews, telephone interviews, and focus group interviews, have been used depending on the purpose of the study. The main research questions have been related to the process or effect evaluations of these projects. The majority of the studies have been carried out with adolescents, as many of the projects receiving grants from the NBHW are targeting adolescents for the purpose of preventing alcohol and tobacco use. In three studies, data have been collected from both adolescents and parents, and two of these are longitudinal studies with adolescents and their parents. Dyads of adolescents and parents are identified and have been followed over the three years of secondary school. All youth surveys have been carried out in a school environment while the questionnaires to the parents have been sent by mail. Municipalities, schools, and organizations across Sweden have participated in the studies. There are many advantages with the partnerships that have been developed between the research team and the project leaders within the NGOs. For example, the large scale of the studies that have been carried out during the six years could not been managed without this cooperation. The project leaders have done much of the practical work locally, such as the dialogue with participating schools and organizations, distribution of questionnaires, and sometimes also feedback to participants.

**Table 5:** Description of the empirical studies, participants, methods, and time period.

		Numbers of participants by school year						Total
		03-04	04-05	05-06	06-07	07-08	08-09	
**I**	**Adolescents Age (school year)**							

Questionnaire	11-12 (5)	238	36					274
	12-13 (6)	283	250					533
	13-14 (7)	257	753	341	203	827		2,381
	14-15 (8)	260	377	799	250	858	826	3,370
	15-16 (9)			277	589		821	1,687
	10-15 (Russia)			647	647			1,294

Focus group	Girls (Verdandi)					15		15
Interview	Members of youth Club (Verdandi)					14		14

	**Total**	**1,038**	**1,416**	**2,064**	**1,689**	**1,714**	**1,647**	**9,568**

**II**	**Parents**							
	**Age of the child (school year)**							

Questionnaire	Pregnant women		585					585
	12-13 (6)		57					57
	12-14 (6-7)		60					60
	13-14 (7)		612			671		1,283
	14-15 (8)			475		656		1,131
	15-16 (9)		78		411		561	1,050
	10-15 (Russia)				580			580
	13-19		33					33
Telephone	School year 9				53			53
interview								

	**Total**		**1,425**	**475**	**1,044**	**1,327**	**561**	**4,832**

**III**	**Actors, Stakeholders**							

Questionnaire	NGO Project leaders	41	37			27	26	131
	Teachers		17					17
	Top athletes	31	16					47
Interview	NGO Project leaders	7						7
	Supervisors		4					4
	Stakeholder Football					32		32

Focus group	Peer supporter, 14-6			20				20
	Girl group leaders				9	5		14
	Youth leaders				15	6		21
e-mail survey	Sports Organizations					7		7

Telephone	Shop owners		27					27
interview								

	**Total**	**79**	**101**	**20**	**24**	**77**	**26**	**327**

**TOTAL**		**1,117**	**2,942**	**2,559**	**2,757**	**3,118**	**2,234**	**14,727**

### What types of research and development have been included?

All projects in the project portfolio had to submit semi-annual and annual reports. These reports were analysis and synthesized into an annual report to the NBHW. This was based on a reporting format using questions for different important elements in the projects as well as key aspects of project management. The preparation of the annual reports included cross-project comparisons with regard to the case study questions, which resulted in some amendments and changes over the years in the research and development activities.

After the decision on potential projects for in-depth studies, planning meetings were convened with the project leaders and managers in the NGOs. Based on the project proposals and joint planning between the project leaders and the researchers, a plan for the in-depth studies was developed. Depending on the evaluation and research questions and available resources the focus, design, process, and outcome measures were set (Table 6). The overall results were positive; ten of the fourteen in-depth studies were completed. One project did not succeed in recruiting high-risk parents to a parental support program (IOGT-NTO Centro). Three projects were only partially completed: one started before the research team was organized, making the evaluation impossible (IOGT-NTO: Dare/Young and King); one was cancelled after a decision by the municipality (SMART Västernorrland); and in one it was impossible to follow up information from policy-makers due to a low response rate (Makalösa föräldrar). Eleven of the in-depth studies started during the first period (Table 4). There were some common research questions such as the effects of the projects. The NGOs wanted their approach to be studied in such a way that, in the event of positive results, the program could be regarded as evidence-based.

**Table 6:** Design, focus, and results of in-depth studies 2003-2009.

	Design	Focus	Completed
**Hassela solidaritet**: Peer Support in School	Repeated cross-sectional	School children	Yes

**IOGT-NTO Centro**: Parental Support	Before-after questionnaire	Parents	No

**IOGT-NTO**: Parents Together	Longitudinal Cluster randomized controlled trial	Parents and their children	Yes

**IOGT-NTO**: Strong and Clear	Longitudinal quasi-experimental study	Parents and their children	Yes

**IOGT-NTO**: Dare/Young and King	Follow-up study	Parents	Partly

**IOGT-NTO:s Juniorförbund**: Junis sisters	Process evaluation	Children Group leaders	Yes

**KSAN**: About small things	Randomized controlled trial	Pregnant women	Yes

**Makalösa föräldrar**: Single Parent with Teenagers	Follow-up study	Group members Policy makers	Partly

**Motgift Gotland**: Antidote Gotland	Process evaluation	Actors in community	Yes

**The National Federation SMART**: SMART Västernorrland	Repeated cross-sectional	Schoolchildren	Partly

**Söder mot narkotika**: Södermalm against Drugs	Process evaluation	Actors in community	Yes

**The Swedish Ice Hockey Association**: School Ambassadors	Process evaluation	Ambassadors Schoolchildren	Yes

**UNF**: Folk Beer Project	Quasi-experimental study	Purchaser	Yes

**Verdandi Tensta Rinkeby**: Get Safe in Tensta Rinkeby	Process evaluation	Actors and Members	Yes

### Effect Studies

Seven projects were considered for evaluation with effect studies, which were planned for all seven projects. However, one project was unsuccessful and two only partially completed due to overly limited implementation. One project was already implemented when the research group was appointed. It was nevertheless possible to plan and successfully complete effect studies even with short-term yearly funding.

#### KSAN "About small things"

The aim of this project was to develop and test an early intervention targeting pregnant women to prevent alcohol injuries in unborn children. The project was developed by the KSAN, an umbrella organization for women's organizations concerned with alcohol and drug issues, and the Swedish Association of Midwives. It was implemented in a maternal health centre in Stockholm. A randomized controlled study was completed with 454 mothers randomly assigned to either receiving an information folder with the message "Pregnancy is not a time for risk-taking" sent to their home after the telephone contact for booking the first visit to the midwife, or getting the folder during their first visit to the midwife. The effects of the intervention were measured by a questionnaire that the pregnant women answered at the maternal health centre before they met with the midwife.

#### IOGT-NTO: Strong and Clear

Strong and Clear is a parental support program targeting parents with children aged 13-16 years. It is a universal program aiming to prevent drinking among adolescents and to maintain parents' restrictive attitudes concerning adolescents and alcohol. The program is manual based and includes thirteen activities during the three years of secondary school. The parents can sign up for the program during the whole period the program is carried out. There are both group and self-administered activities divided into four types: parent meetings, family dialogues, friend meetings, and family meetings. The program was implemented in six schools.

The research program includes the effect study, which was designed as a longitudinal quasi-experimental study, and studies of parental attitudes and behaviour with regard to adolescents and alcohol [78] as well as reasons for non-participation [73]. In the longitudinal study, 706 children and 613 parents participated in the baseline questionnaire, which was followed by repeated data collection in the two following school years.

#### IOGT-NTO: Parents Together

The program Parents Together consists of three parents' meetings during three years in secondary schools. The intention is to motivate the class parents to come to an agreement on the following issues: "We enforce the 18-year limit for alcohol; We will not provide each other's children alcohol; We will get in touch with each other if we see a child we know who is not sober, is behaving badly, or is out at times and places where we would not want our own children to be."This agreement is used to strengthen the cooperation among parents. The idea is that this will make a difference with respect to the children's alcohol use. A parent-teachers meeting is held each year to update the agreement.

**Figure 3:**
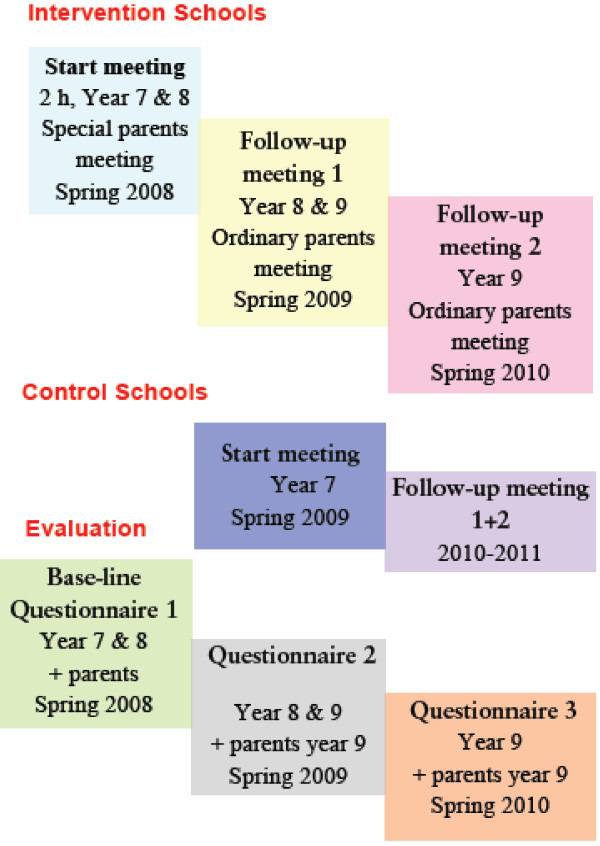
**Design of the intervention and evaluation of the program Parents Together**.

The design of the study is a cluster randomized controlled study in Swedish secondary schools with seven intervention and six control school. The study included almost 2000 pupils and their parents. The program Parents Together was carried out over three years in the seven intervention schools with a start in both school years 7 and 8 (Figure 3). The six control schools have been offered the program for parents whose children are in year 7 in the spring 2009 and the program will follow in the years 2010 and 2011. To reveal effects of the program the evaluation also includes a questionnaire about the prevention work in schools and implementation reports. The non-governmental organization IOGT-NTO is responsible for the program and the implementation in the seven intervention schools. To maintain the cooperation between the thirteen schools, the NGO, and the research team, an agreement has been signed. The agreement includes information about the responsibilities of each party such as that the researchers should the results of surveys, within six months after the data collection, are published on the website.

#### IOGT-NTO Centro: Parental Support

This project was planned to include before and after questionnaires to high risk parents. However, the project did not succeed in attracting this type of parents to the program.

#### Hassela solidaritet: Peer Support in School

This NGO works with training and assisting school children to be peer supporters in their own school. The aims of the project are to prevent social exclusion by reducing teenage alcohol consumption, experimentation with drugs, and bullying through peer support in schools, and to promote a school that is a positive, creative, and stimulating workplace for all. The program was first implemented in one part of the school, and was planned to be extended to the whole school. Subsequent implementation in a second school was planned. However, this extended implementation was only partly carried out due to limited resources. The evaluation included focus group interviews with peer supporters and repeated cross-sectional questionnaires to schoolchildren in school years 7-9 in the two schools.

#### National Federation SMART: SMART Västernorrland

The main objective of this NGO is to prevent or postpone alcohol, tobacco, and other drug use among children through positive reinforcement and signing of contracts. The parents sign the contract together with their child. The content of these contracts varies between local organizations. The membership gives the child positive benefits reinforcing positive behaviours. The program was implemented in a Swedish county, Västernorrland. The evaluation plan included an effect study among schoolchildren in Kramfors, a study of parents, and an interview of stakeholders in the county. The program was cancelled by the municipality of Kramfors with negative consequences for the effect study, which had been planned as a repeated cross-sectional study of schoolchildren in years 4-9. Data was collected with questionnaires and during the three years, 2,052 children answered the questionnaire. The research team decided to implement the evaluation as planned even if there was no intervention the third year.

#### The Swedish Youth Temperance Movement (UNF): Folk Beer Project

The Non-governmental organization UNF is a politically and religiously independent organization. They are a sister organization to IOGT-NTO (The Swedish Temperance organization), which is a part of the International Organization of Good Templars. All members are between 13 and 25 years of age. To be a member you have to be a teetotaller. The activities are of different kinds, for example arranging theatrical performances, discos, cafés, study circles, and a large number of courses. Besides dealing with alcohol regulations and politics regarding alcohol, they also work with international exchange and democracy issues. Their vision is a democratic and socially responsible world free from drugs. Although they are politically independent, their task is to act politically in letting the politicians know which issues are important to them. UNF has an almost 40-year history of conducting underage alcohol purchase attempts.

In 2003 UNF applied for funding for a new idea. They wanted to compare two different strategies that included underage purchase attempts. The first was an elaboration of their earlier method, which meant confronting the media with the results of the purchase attempts, reporting the check-out clerks who sold them beer to the police, and informing the municipalities of which stores that sold beer to minors. The other method was based on the idea to actively seek cooperation with the retail grocery sector, the municipality's alcohol administrator or drug coordinator (the municipalities are organized differently), the police, and the labour unions. The evaluation program was designed as a quasi-experimental study and as a follow up of the alcohol legislation concerning the sale of beer to minors in Sweden [75] and of the effect of different strategies to influence the shops to comply with the law [74].

### Studies of Process and Implementation

Seven of the in-depth studies focussed on the working process in the projects. Three projects were community-based and had a clear geographical area where the programs were implemented. Motgift Gotland was an alliance for preventing the use of narcotics on the island of Gotland. Söder mot Narkotika was also an alliance against narcotics in a central district (Södermalm) of the capital Stockholm. A broad range of agencies and organizations collaborated in these efforts. A third community-based project was run by Verdandi Tensta Rinkeby. The three community-based projects were studied during 2005-2006 and included interviews with stakeholders and actors in the projects. A lesson learned is that community-based prevention needs to have broad support and cannot depend heavily on individual project leaders.

#### Verdandi: Get safe in Tensta - Rinkeby. Meet us!

An in-depth analysis was made of the third project in order to uncover their successful strategy. Verdandi, founded in 1896, is a Swedish workers' organization striving for social justice and a society free from alcohol-related injuries. From the very beginning, Verdandi - as an independent organization within the workers' movement - has aimed to improve people's social and financial situations. Today's aim is to analyse the development of society through the experiences and voices of those who are not heard otherwise. People of all ages, in all parts of the country, may participate in Verdandi's activities, which are quite different from place to place since they are based on local needs. According to Verdandi, without a local angle, the organization would soon lose touch with reality as well as lose credibility and members.

Verdandi runs activities for youth. The project includes support for children both in school and after. The youth in the organization can use a facility in the neighbourhood in their leisure time. Youth activities have focused on "the young leading the young" and the project has demonstrated young people's ability to organize and run a rewarding activity in the evenings and on weekends. The aim of this prevention program is to empower young people in their daily lives and help them empower their friends. This, according to the organization, contributes to young people avoiding drugs, and the neighbourhood has become calmer and safer. The activity has a bottom-up nature and the youth are involved in the planning. They have the opportunity to develop activities and thereby affect their daily lives. Among the success factors, according to the in-depth study, are: confidence in the organization, equality, youth involvement and power, memberships, support from the parents, training of leaders, common norms and roles, volunteer work, easily accessible premises, and a leadership that facilitates democratic processes.

#### IOGT-NTO: Dare/Young and King

This program is a redesigned version of the American program DARE [79], which was implemented before the research group was appointed. However, an adult education component, Young and King, aiming to strengthen parents was implemented and a follow-up study was completed of this component of the program.

#### IOGT-NTO:s Juniorförbund: Junis sisters

In this project, groups of schoolgirls in years 5 and 6 are organized with the objective to strengthen their self-esteem and promote meaningful leisure activities and thereby delay the onset of alcohol consumption by the girls. The evaluation focused on the group leaders, who were interviewed in focus groups. A lesson learned is that special effort must be put into recruiting and assisting group leaders to achieve sustainable programs.

#### Makalösa föräldrar: Single Parent with Teenagers

The project consisted of two main parts. One part focused on improving the knowledge about how it is to be a single parent with a teenager in the family. A survey of single parents was done in a part of Stockholm and a small newsletter was produced. The other part included self-help groups for single parents and summer camps. The evaluation of the self-help groups consisted of follow-up questionnaires to participants. An unsuccessful part of the evaluation was the follow-up of the newsletter, which was well planned and properly designed. It was not possible to get feedback from policy-makers on the publication, which may be due to lack of awareness of the publication and its contents.

#### The Swedish Ice Hockey Association: School Ambassadors

The project aimed to train top athletes to become school ambassadors in order to influence the attitudes of schoolchildren and give them the opportunity to try out ice hockey. Moreover, the project was also an attempt to improve the collaboration between schools and top ice hockey clubs. In the second year the specialized ice hockey secondary schools were included in the program. The evaluation consisted of following up the training of the athletes and studying the work of the secondary school ice hockey players by means of a questionnaire to schoolchildren.

### How can a trustful partnership develop between practitioners, national agencies, and researchers?

In order to promote the development of a partnership a series of measures were implemented (Table 7). All project leaders were invited to regular meetings, which were held in Örebro as well as in Stockholm, Gothenburg, and Malmö. The agenda included presentation of project plans, information from the NBHW, and the research and development activities by the research team. Thematic lectures and discussion on issues such as the art of project management, measures to reach target groups, media advocacy, Internet as a tool for prevention, and planned communication were held at different meetings. The main objective of these meetings was to promote exchange of experiences and learning in order to strengthening the quality of the implementation of the projects as well as networking. Moreover, the systematic bi-annual and annual reports were introduced and discussed.

**Table 7:** Implemented measures to promote partnerships between NGOs and researchers at Örebro University

Measures	Focus	Participants	Extent
Meetings with project leaders	Documentation and thematic discussions	All	16

Project Dialogues and Consultations	Strengthening project implementation and evaluation	Optional	3-5 per year

Competence building	Formal Academic Training Supervision in Groups	Optional Optional	2004-5 Yearly

Support for Documentation	Web-based reporting system	All	2 per year

In-depth Studies	Process and Effect Studies Including Feedback to NGOs	Selected projects	14

National Conferences	Reflection on Prevention - Evidence and Collaboration	All	2006, 2008, 2010

Depending on the needs of the different projects special project dialogues and consultations were held between individual projects, or a small group of similar projects, and the research teams. The results of these meetings ranged from refinement of project ideas to long-term collaborations. All in-depth studies started with such meetings. The competence development took different forms. In the first period an academic training program in alcohol and drug prevention was offered to the project leaders, of whom about 10 participated. Supervision in groups was implemented in three groups during the first two years, and thereafter one or two groups were run by independent supervisors annually. During 2009 the research team arranged more project leader meeting including training in project management as an alternative to supervision.

The in-depth studies were also an important measure to foster the partnership between the NGOs and the research team. Due to available resources, more extensive process and effect evaluation activities could only be implemented in a limited number of projects. Many more projects asked to be the focus of in-depth studies than the fourteen that were initiated.

The research team together with the NBHW arranged a national conference Reflections on prevention - Collaboration for better alcohol and drug prevention. Conferences were arranged in the spring of 2006, 2008, and 2010. Among the key issues discussed at the first conference were the role of parents in prevention, adolescents, community-based approaches, and supply-reducing initiatives. The second conference also discussed the role of civil society and how to promote more effective cooperation among the different stakeholders. The third conference focussed on evidence and evidence-based practice, which have received increased attention in Sweden in many sectors of society. A main emphasis has been setting the context for reflection and sharing of experiences among the participants at the conferences; therefore a series of seminars with project presentations and panel discussions have been part of the conferences. Moreover, plenary sessions as well as theatrical performances further set the stage for professional dialogues on alcohol and drug prevention. The conferences have been well received and have attracted actors from different sectors of society as well as national agencies and NGOs.

**Figure 4:**
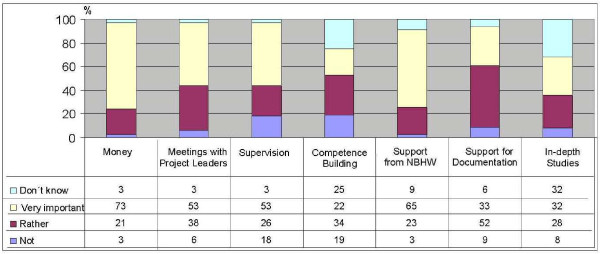
**Assessment by project leaders of measures to improve collaboration between NGO and research in 2006**.

In the annual reports the project leaders also assess the implemented measures by the researchers. These have guided the future efforts of the research team. As an example, the assessments made in January 2005 are presented in Figure 4. The financial support was very important, followed by the support from the NBHW, the project leader meetings, and the supervision. One third of the project leaders regarded the support for the documentation as very important. The academic training in alcohol and drug prevention was regarded as very important by one fifth of the project leaders, which is a high proportion given that only a small group participated in the distance education course. Only eight projects were at that time included in the in-depth studies, nevertheless one third of the project leaders reported this measure as very important. The case study data bank includes information for questionnaires, interview and other data sources for the assessment of the implementation of the research strategy.

The research strategy has been successfully implemented despite the fact that only some projects were running more than one year while new projects and project leaders are included every year. The first two years a focus in the meetings with the project leaders was on the in-depth studies which were presented by the organisations and the researchers. Then the focus changed to addressing common concerns among the project leaders such as how to reach target groups, use of Internet, different type of prevention projects and mass communication skills. The presentations from the research teams were more concentrated to the national conferences that were organized bi-annually. The networking between the projects also resulted in new applications jointly by two organisations.

An important element was the relationship between the NBHW and the research team at Örebro University. During this period the NGO portfolio was managed at the NBHW by the same senior official. However, the department director changed three times during this period. The members of the working committee also changes over the years. The chairman was the same during all years. The support to the research and development activities was nevertheless maintained and also renewed for another year. The continuity with regard to persons seems to be very important for such an endeavour as included in this case study.

## Discussion - towards practice-based research for alcohol and drug prevention

The integration of the research and development component into the support from the NBHW resulted in a unique possibility to do comparative studies involving, among other things, project management and implementation as well as project results. The measures to promote a partnership for practice-based research also improved the quality and success of the different projects. A few of the in-depth studies were unsuccessful due to factors hindering the implementation, and in several cases these factors were related to a lack of resources on the part of collaborating partners in the municipalities or other organizations.

The research strategy has been based on the overall aim to contribute to the evidence base for alcohol and drug prevention, an emphasis that this field shares with health promotion, prevention in general, and social work [2-8]. The current development of practice-based research will give more relevant knowledge and our research program attempts to be a part of this trend. Moreover, the utilization of research results may also be improved if studies on efficacy, effectiveness, and dissemination are promoted [18]. The strategy that the NBHW developed in this program of governmental support to NGOs was an attempt to bridge this gap as described by Nutbeam [10,11]. This challenge for agencies to respond to the push from the funders and pull from the communities has been noted by Green and Mercier [23] and the public health researcher also needs to leave the university campus to get involved in more practice-based research. Our research program has developed along such lines.

The research strategy includes the use of qualitative, quantitative, and mixed methods. This means that data collection and data analysis are done using guidelines for these three traditions. The challenge is most apparent with regard to inference and integration. In the stages of inference in a study, quality issues such as internal and external validity in the quantitative approach and aspects of credibility, confirmability, and transferability in the qualitative approach are pertinent. Integration is the mixed-methods approach of working across strands and using meta-inferential issues related to the integration of findings and inferences from the two strands. Here design quality, interpretative rigour, and inference transferability have been proposed as indicators of quality [70]. In this research strategy case study, the set of research entities changed each year due to the funding of applications from NGOs by the NBHW. The present study covered a six-year period, and the stages of inference and integration were completed yearly in the preparation of the annual reports to the NBHW.

The research strategy also includes elements of participatory research. The organizations were involved in developing the main research questions in the in-depth studies. Sometimes the organizations also assisted in collecting questionnaires from school children; in making participatory observations, as in the studies of beer purchasing by minors; or in providing feedback to school staff and target groups, as in the parental support programs. Moreover, the organizations also played a role in discussing preliminary results as part of a validating process for the empirical studies. These discussions never changed the interpretations of the findings but often gave more insight into the noble art of implementation of preventive programs. Nevertheless, as in other research programs, a number of methodological challenges had to be dealt with. The resources were limited, which gave room for only a small number of in-depth studies. Therefore the research strategy included additional elements such as support to documentation as well as support to the project leaders in meetings and management training. The selection of these studies was mainly done by the steering committee at the NBHW. The research team developed a proper design for these studies based on the assessed potential for a successful implementation and possible options given the resources available for effect or process studies. Then the choice of methods for data collections was reviewed and target groups for the evaluation research selected. In this process the best choice from an academic point of view was often not possible due to limited staff and other resources. Nevertheless, the research program resulted in data collected from 9,568 children, 4,832 parents and 327 actors or stakeholders. Moreover, it was possible to carry out two large longitudinal studies of children and their parents in this research program. Even if the funding was granted annually, it was possible to think and plan on more long-term basis.

A broad range of organizations received project funding from the NBHW. Although the largest number of projects was run by the nine alcohol and drug organizations, the alcohol and drug prevention was successfully integrated into a range of organizations with other main objectives. Moreover, the project leaders also came from different societal sectors. This was an intended effect of the governmental initiative to strengthen the alcohol and drug prevention in Sweden. This led to another methodological challenge caused by the fact that the programs were so different. The research team developed questionnaires with common modules that could be used in different evaluations thus giving them access to data from schoolchildren and parents in different contexts and programs. This made it possible, for instance, to study the reasons for non-participation in parental support programs [73]. Another added value related to this was the possibility to organize a study of project management through a special study of the project leaders, which was integrated into the overall design of the support from the NBHW.

A challenge for the research team was that the funding for the research as well as for the alcohol and drug projects was decided annually by the NBHW. However, the research was planned with a longer time period in mind, which has actually led to a research program that has been running more than six years. A more long-term grant would have been beneficial for the development of a partnership between the NGOs and the research team. In order to overcome this barrier a multi-year agreement has been signed for the newer in-depth studies, but it was still signed on the condition of renewed funding the following year. Nevertheless, a trustful partnership was developed between all three partners: the practitioners in the NGOs, the national agencies, and the researchers. In many cases the planning and implementation were done jointly, dividing the responsibilities according to skills and keeping the roles clear and feasible to complete successfully. The validity of the results was also a major concern as well as an emphasis on a participatory approach to the research process.

Ethical concerns were very important, as stipulated by Swedish law. The effect studies were assessed by the regional research ethics boards. However, it is also important to analyse if the NGOs have vested interests in the research process. Government agencies as well as NGOs can also have a vested interest in scientific research, such as when science is misused to benefit a particular political agenda, ideology, or favoured interest group [80]. However, the problem of vested interests is more dangerous when key parts of the government sector are in conflict over their public health responsibilities; for instance health sector engagement in partnership arrangements with addictive consumption industries (particularly alcohol, tobacco, and gambling) entails too many risks [81]. In our case there have been shared visions and objectives between the government agency and the NGOs, which guided the developmental activities as well as the research work. Moreover, the division of responsibilities between the NGOs and the researcher was important. The NGOs had the responsibility for developing the proposals, conducting the interventions, and implementing the preventive programs or initiatives. The researchers had the responsibility for planning the effect evaluations after consulting with representatives of the NGOs, as well as for implementing the research components, analysis, and reporting of results, including dialogues about the outcomes, and presenting the findings for the NGOs.

The organization of the research program under the auspices of public health science at Örebro University was natural as the principal investigator holds a professorial chair in public health there. During the first two years, other members of the research group were formally employed by an NGO but worked at the university campus in Örebro. All members of the research team were subsequently offered employment at the university, giving the research team a formal independence from all NGOs.

The addiction research centres have mandates that are broader than the present research program. The centre in Michigan has the mission to develop new knowledge about the cause, course, and consequences of substance use disorder and to train the next generation of researchers [28]; and the Canadian centre in British Columbia to create an internationally recognized centre distributed across BC that is dedicated to research and knowledge exchange on substance use, harm reduction, and addiction [27]. The Swiss institute is primarily involved in collecting alcohol-related information and making it available to professionals and the general public. The Swiss Institute will continue to monitor substance use, while stepping up its prevention research activities and ensuring that it is able to react promptly to emerging phenomena [25]. Our small research team is attempting to fill a gap in knowledge about the NGO alcohol and drug prevention efforts as these offer unique opportunities [82].

The research strategy was successful in establishing prevention research for alcohol and drug prevention by NGOs, which previously had been lacking in Sweden. Moreover, added value came from having meetings for project leaders, and the capacity building led to new innovative collaboration between different NGOs, which resulted in new applications for funding and successful implementation of new initiatives. The administrative support for improving the documentation of the implementation and progress of the projects was also recognized as beneficial for the practitioners and the national agency as well as the researchers. The best approach is always transparency and discussion, disclosure and debate [83,84].

A weakness in the research strategy was that the funding was not sufficient for more than a limited number of in-depth studies. The role of *researchers-as-technical advisors *was suitable for the fostering of a trustful partnership for research and development. The independence of the NGOs was regarded as important for the momentum in the project implementation. It was beneficial because it gave the research team opportunities to address other issues. From a strictly research point of view it would have been of interest to see what could be achieved by *researchers-as-designer*, but this would have been very costly and all funds allocated to the integrated research activities would have been consumed by just one project. In other words, the present research strategy can be regarded as cost-effective.

The overall strategy for research and development includes capacity building for both the practitioners in the NGOs and the research team, and two doctoral dissertations will be finalized during the coming year. The NBHW has also noted that, given the limited duration of funding, this organization of knowledge development - as an integral part of the support to NGOs - is beneficial, which is indicated by the annual renewal of the contract with Örebro University. Moreover, the much more extensively funded projects in municipalities, regions, and counties still lack this strategic element. At present there is a trend that some larger governmental grants are given to such parties, but a mandatory linkage to universities for research is included in the call for proposals. This could lead to similar forms of trustful partnerships as found in the present research strategy case study.

The in-depth studies in this research strategy varied in content, design, and size. A common element was that they were program-driven and not research-driven interventions [9]. This may give the studies improved external validity [54]. The research strategy aimed at improving the evidence-base for alcohol and drug prevention. In our case this has meant using qualitative, quantitative, and mixed methods, as well a variety of designs to answer questions in practice-based settings. Including feedback and dialogue with the NGOs has further contributed to sustainable AD prevention in different settings. The missions of the NGOs differ, but the AD prevention has been included as an essential part of their activities, which in many cases meant that AD prevention has received increased priority. Moreover, the integrated research program has also been seen as beneficial and important for the organizations, which often wanted their programs to be recognized as evidence-based. Therefore, the demand for research by the NGOs is larger than what we can supply at present. This is a challenge to the funding agencies as well as research bodies. The addiction research centres seem to nurture creativity but often lack the networks and priorities for preventive research. It is important to go beyond the notion that a lack of evidence for a program is necessarily a sign of a lack of effectiveness. Therefore, practice-based research and collaboration between decision-makers, national agencies, NGOs, local authorities and researchers is needed. Using a combination of different and interactive measures it was possible to over the years built a trustful partnership between these parts. This research strategy case study shows that it is possible even in such a dynamic field as alcohol and drug prevention in NGOs where the organisations are competing for grants from the NBHW. There are added values in supporting a research group assigned to a project portfolio instead of having a series of smaller independent evaluations.

## Conclusions

This research strategy case study shows that it is possible to integrate research into alcohol and drug prevention programs run by NGOs, and thereby contribute to a more evidence-based practice. A core element is developing a trustful partnership between the researchers and the organizations. Competence development is necessary for developing evidence for policy and practice. Given research groups assignments to address the knowledge development issues is better than having minor evaluation in individual projects. Moreover, the funding agency must acknowledge the importance of knowledge development and allocating resources to research groups that is capable of cooperating with practitioners and NGOs.

## Competing interests

The authors declare that they have no competing interests.

## Authors' contributions

The four authors of the manuscript are presented in alphabetical order and their shares of the responsibility for the paper are equal. CE is the principal investigator for the research program integrated in the NBHW support to NGOs for alcohol and drug prevention. CE, ML, and CP were involved in all aspects of the program as well as this study. SG was involved in the planning, project implementation, and writing of the section on NGOs. All authors read and approved the final manuscript.
